# The impact of genomic relatedness between populations on the genomic estimated breeding values

**DOI:** 10.1186/s40104-018-0279-4

**Published:** 2018-08-16

**Authors:** Peipei Ma, Ju Huang, Weijia Gong, Xiujin Li, Hongding Gao, Qin Zhang, Xiangdong Ding, Chonglong Wang

**Affiliations:** 1 National Engineering Laboratory for Animal Breeding, Laboratory of Animal Genetics, Breeding and Reproduction, Ministry of Agriculture, College of Animal Science and Technology, China Agricultural University, Beijing, 100193 China; 2 Department of Animal Science, School of Agriculture and Biology, Shanghai Jiao Tong University, Shanghai, 200240 China; 3 Gènes DIFFSUION, 3595 Route de Tourna, 59500 Douai, France; 4 Center for Quantitative Genetics and Genomics, Department of Molecular Biology and Genetics, Aarhus University, Tjele, Denmark; 5 Key Laboratory of Pig Molecular Quantitative Genetics, Institute of Animal Husbandry and Veterinary Medicine, Anhui Academy of Agricultural Sciences, Hefei, 230031 China

**Keywords:** Genomic prediction, Genomic relationship, Joint population prediction

## Abstract

In genomic selection, prediction accuracy is highly driven by the size of animals in the reference population (RP). Combining related populations from different countries and regions or using a related population with large size of RP has been considered to be viable strategies in cattle breeding. The genetic relationship between related populations is important for improving the genomic predictive ability. In this study, we used 122 French bulls as test individuals. The genomic estimated breeding values (GEBVs) evaluated using French RP, America RP and Chinese RP were compared. The results showed that the GEBVs were in higher concordance using French RP and American RP compared with using Chinese population. The persistence analysis, kinship analysis and the principal component analysis (PCA) were performed for 270 French bulls, 270 American bulls and 270 Chinese bulls to interpret the results. All the analyses illustrated that the genetic relationship between French bulls and American bulls was closer compared with Chinese bulls. Another reason could be the size of RP in China was smaller than the other two RPs. In conclusion, using RP of a related population to predict GEBVs of the animals in a target population is feasible when these two populations have a close genetic relationship and the related population is large.

## Short communication

Since genomic selection (GS) was first described by Meuwissen et al. [1], with the constantly decreasing genotyping cost, this technology has revolutionized breeding of both livestock and crops in the last few years. The size of reference population (RP) and the relationship between the reference and candidate population were reported to be the important factors affecting accuracy of genomic prediction [2–4].

The advantage of using GS has been limited due to limited size of RP. Firstly, a low number of progeny-test proven bulls were available in each country especially in countries which mainly relied on importing bull semen from the other countries, e.g. China [5]. Secondly, it is not economically feasible to genotype all the animals as RP since the contribution of the cows may be less than the cost for genotyping them [6]. To gain accuracy of GEBV, two strategies were used in practice. One strategy is to combine the reference data from several countries. The other one is to use the RP from a commercial institute e.g. CDCB (https://www.uscdcb.com/what-we-do/genomics). However, it was reported that the relationship between RP and candidate individual was a crucial factor for prediction accuracy in genomic prediction [7, 8]. Therefore, it is necessary to investigate the relationship between populations before applying these strategies.

The objectives of this study were 1) to investigate the correlations between genomic estimated breeding values (GEBVs) for French bulls using Chinese, French and American RP separately; 2) to explore the reasons led to different GEBVs, by analyzing the linkage disequilibrium (LD) phase persistence, genetic relatedness, and population structure among French, American and Chinese populations.

## Materials and methods

### Data

A total of 122 French bulls were used as test set in this study. The GEBVs of milk yield, fat percentage, protein percentage, confirmation and feet_legs evaluated using American RP and French RP separately was provided by Gènes DIFFSUION. The GEBVs of these 122 French bulls using Chinese RPs were estimated in this study. The Chinese RP consisted of 1,568 Chinese cows with both genotype and phenotype. De-regressed proof (DRP) was used as the response variable for genomic prediction in this study. Genotypes of 270 French bulls, 270 American bulls and 270 Chinese bulls were used to compare the relationship among three populations. These 270 French bulls were the progenies of the imported French bulls and cows. So did the American bulls. The Chinese bulls were randomly selected from the native population. All the animals were genotyped using Illumina Bovine SNP50 BeadChip (Illumina, San Diego, CA, USA). After deleting SNPs with a minor allele frequency smaller than 0.01, 45,404 SNPs on 29 autosomes were retained.

### Model

GBLUP [9] was used for prediction of GEBV using Chinese RP. The model is as follows:

$$ \boldsymbol{y}=\mathbf{1}\mu +\mathbf{Zg}+\mathbf{e} $$

where ***y*** is a vector of DRP from Chinese population, *μ* is the overall mean, **g** is a vector of GEBV, **1** is a vector of ones, **Z** is the design matrix for linking **g** to ***y***, and **e** is a vector of the random residuals. Random effects were assumed to be normally distributed as **g**~N(**0**,$\mathbf{G}{\sigma}_g^2$) and **e**~N(**0**,${\mathbf{I}\sigma}_e^2$),where ${\sigma}_g^2$is the additive genetic variance, ${\sigma}_e^2$ is the residual variance, **G** is the genomic relationship matrix constructed with all the markers using the formula **G** = **MM**^′^/ ∑ 2*p*_*i*_(1 − *p*_*i*_) [9]. The genotypes in M were coded as 0, 1, and 2 for A_1_A_1_, A_1_A_2_ and A_2_A_2_ and then centralized by subtracting 2*p*_*i*_ [9], where *p*_*i*_ was the allele frequency of A_2_ and was calculated based on the genotypes from the individuals used in the model. DMU package [10] was used to estimate variance components and obtain solutions of the mixed model equations.

### Validation of genomic predictive ability

The Spearman’s rank correlation coefficient between GEBVs predicted using different RPs was used as a measurement of concordance of GEBVs. The correlation coefficient between GEBVs evaluated from Chinese RPs and from French RPs was named as COR_CF_. Accordingly, COR_CA_ was used for that between Chinese RPs and American RPs and COR_FA_ for that between French RPs and American RPs.

### The measurement of relatedness between different populations

To examine the genetic relatedness between different RPs, three measurements of genetic distance were performed for 270 French bulls, 270 American bulls and 270 Chinese bulls: 1) the persistence of LD phase between two populations. It was calculated as the correlation of linkage disequilibrium (*r*^2^) of adjacent marker pairs on each autosome [11, 12]. The persistence of LD phase between each pair of these three populations was named PER_CF_, PER_CA_, PER_FA_. 2) the number of pair of related individuals between different populations. All pair-wise relationship can be classified as monozygotic twins, 1^st^-, 2^nd^- or 3^rd^- degree relatives by the estimation of kinship coefficients using Kinship-based INference for Genome-wide association study (KING) software package [13]. 3) the principal components (PCs) of marker genotype data. Principal components analysis (PCA) was performed on genotype using KING [13]. We used the plot of PC2 against PC1 as the description of genetic similarity among three populations.

## Results

### The comparison of genomic prediction using different RPs

The spearman’s rank correlation coefficient between GEBVs using RP from two of three countries is shown in Table 1. For all traits, the correlation between GEBVs using French RP and using American RP (COR_FA_) is much larger than the correlation between GEBVs using Chinese RP and using American RP (COR_CA_) or French RP (COR_CF_). COR_FA_ for fat percentage achieved the highest (0.862) while COR_CA_ for milk yield was the lowest (0.060). COR_CF_ ranged from 0.133 (for feet_legs) to 0.442 (for conformation). COR_CA_ was similar as COR_CF_ and ranged from 0.060 (for milk yield) to 0.420 (for protein percentage). COR_FA_ was much larger than COR_CF_ and COR_CA_ and ranged from 0.472 (for feet_legs) to 0.862 (for fat percentage).

**Table 1:** Spearman’s rank correlation coefficient between GEBVs evaluated using different RP

Trait	COR_CF_^a^	COR_CA_^b^	COR_FA_^c^
Milk yield	0.157	0.160	0.752
Fat percentage	0.162	0.167	0.862
Protein percentage	0.403	0.420	0.805
Conformation	0.442	0.448	0.643
Mammary system	0.359	0.402	0.765
Feet_legs	0.233	0.308	0.472

^a^The correlation between GEBVs estimated using Chinese RP and using American RP

^b^The correlation between GEBVs estimated using Chinese RP and using French RP

^c^The correlation between GEBVs estimated using French RP and using American RP

**Fig. 1:**
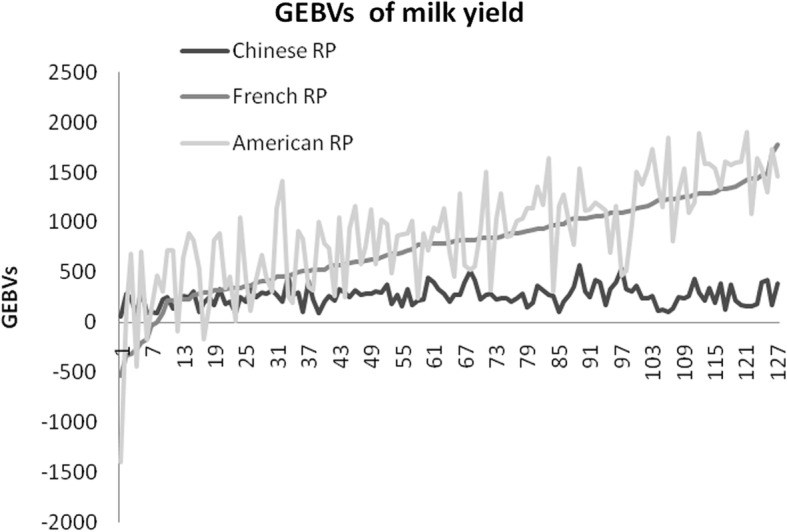
The genomic estimated breeding values (GEBVs) of milk yield for 122 French bulls estimated using different reference population (RP)

The plot of GEBVs of milk yield using different RPs is presented in Fig. 1. The trends of GEBVs using American RP and French RP are similar while the trends of GEBVs of using Chinese RP are relative different from the GEBVs using the other two RPs.

### LD and persistence of LD phase

The LD of each chromosome from each population and persistence of LD phase (PER) between populations are shown in Table 2. The mean *r*^2^ of adjacent SNP pairs within each chromosome ranged from 0.13 (chromosomes 27 and 28) to 0.19 (chromosomes 6 and 14) for Chinese RP, 0.14 (chromosomes 27 and 28) to 0.20 (chromosomes 6 and 14) in both France and USA RPs. The mean *r*^2^ across all chromosomes were 0.16 in China and 0.17 in France and USA. The persistence of LD phase between France and USA RPs was apparently higher than that between China and the other two countries. The PER_CF_ ranged from 0.893 of chromosome 28 to 0.959 of chromosome 14. The PER_CA_ ranged from 0.931 of chromosome 9 to 0.973 of chromosome 14. The PER_FA_ ranged from 0.942 of chromosome 19 to 0.974 of chromosome 29.

**Table 2:** Linkage disequilibrium (LD) of adjacent markers for each *Bos Taurus* autosome (BTA)

BTA	No. of SNPs	Mean *r*^2^			Persistence^a^		
		China	France	USA	China- France	China-USA	France-USA
1	3431	0.18	0.19	0.19	0.934	0.952	0.968
2	2829	0.17	0.18	0.17	0.930	0.952	0.962
3	2550	0.17	0.19	0.18	0.920	0.946	0.950
4	2570	0.16	0.17	0.17	0.909	0.933	0.948
5	2271	0.15	0.16	0.16	0.926	0.953	0.968
6	2575	0.19	0.20	0.20	0.934	0.954	0.966
7	2352	0.17	0.19	0.18	0.924	0.944	0.961
8	2430	0.17	0.18	0.18	0.912	0.944	0.949
9	2095	0.16	0.17	0.17	0.898	0.931	0.960
10	2206	0.17	0.19	0.19	0.927	0.938	0.955
11	2295	0.16	0.17	0.16	0.930	0.957	0.966
12	1773	0.15	0.16	0.16	0.920	0.947	0.958
13	1850	0.16	0.17	0.17	0.907	0.954	0.960
14	1831	0.19	0.20	0.20	0.959	0.973	0.972
15	1762	0.15	0.16	0.15	0.923	0.941	0.967
16	1726	0.17	0.18	0.19	0.926	0.954	0.948
17	1600	0.16	0.17	0.17	0.922	0.944	0.957
18	1376	0.16	0.17	0.16	0.930	0.944	0.965
19	1420	0.15	0.16	0.16	0.905	0.954	0.942
20	1568	0.17	0.19	0.18	0.919	0.949	0.966
21	1483	0.16	0.17	0.17	0.942	0.964	0.966
22	1324	0.15	0.16	0.15	0.934	0.959	0.968
23	1092	0.14	0.16	0.15	0.921	0.949	0.968
24	1312	0.16	0.17	0.16	0.932	0.959	0.969
25	1004	0.15	0.16	0.16	0.926	0.933	0.972
26	1116	0.15	0.17	0.16	0.904	0.943	0.963
27	981	0.13	0.14	0.14	0.932	0.945	0.967
28	981	0.13	0.14	0.14	0.893	0.939	0.955
29	1087	0.14	0.15	0.15	0.945	0.965	0.974
Mean	52,890^b^	0.16	0.17	0.17	0.924	0.949	0.962

^a^The correlation of *r*^2^ of adjacent SNP pairs between two populations

^b^Sum over 29 autosomes

### The kinship coefficients and classification of all pair-wise relationship

The number of pairs of related individuals in each relationship group which was determined by KING software was listed in Table 3. There was 1 pair of individuals in 1^st^-degree, 1 in 2^nd^-degree and 596 in 3^rd^-degree based on genomic relationship between Chinese population and French population. Based on genomic relationship between Chinese population and American population, there were 2 pairs of individuals in 1^st^-degree, 0 in 2^nd^-degree and 1,174 in 3^rd^-degree. Compared with genomic relationship between Chinese population and French population or American population, there were much more pairs of individuals in 1^st^, 2^nd^ and 3^rd^ degrees based on genomic relationship between French population and American population, which meant there were more related individuals in these two populations.

**Table 3:** The number of pairs of related individuals between different populations

Relationship	Criteria	No. of pairs between CF^a^	No. of pairs between CA^b^	No. of pairs between FA^c^
MZ twin	[0.354,]	0	0	0
1^st^-degree	[0.177,0.354]	1	2	32
2^nd^-degree	[0.0884,0.177]	1	0	1057
3^rd^-degree	[0.0442,0.0884]	596	1174	4447
Unrelated	[,0.0442]	71,492	71,724	66,554
Total		72,090	72,900	72,090

^a^Chinese population and French population

^b^Chinese population and American population

^c^French population and American population

### The principal component analysis (PCA)

**Fig. 2:**
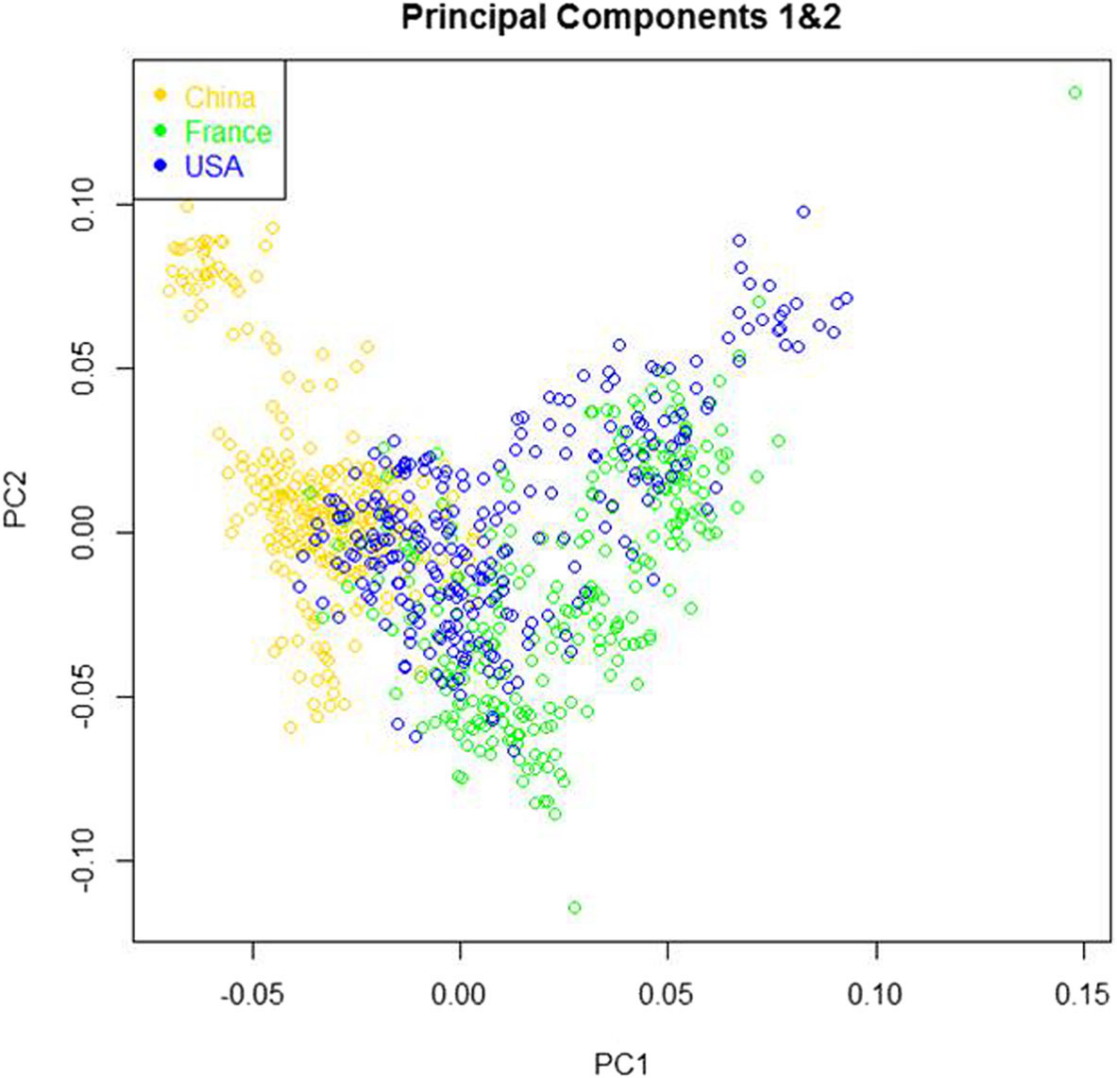
The principal components of marker genotype data.The first principle component (PC1) versus the second principle component (PC2) calculated using marker genotype data

Figure 2 illustrates that the relationship between French population and American population was closer than the relationship between them and Chinese population.

## Discussion

In this study, we investigated the difference on GEBVs for French Holstein bulls using references from different countries. The genomic relatedness between different populations were investigated to illustrate the results. The results showed that the correlation between GEBVs estimated using French RP and using American RP was higher than the correlation between GEBVs estimated using Chinese RP and French/American RP. The LD phase persistence analysis, kinship coefficients and the PCA showed that the relationship between French population and American population was closer than that between Chinese population and American or French population.

For combined RP, a close relationship between populations reflects a similar LD structures among populations which enabled the joint prediction feasible. Lund et al. [14] used European Holsteins as joint reference to predict Nordic Holstein, Dutch Holstein, French Holstein and German Holstein and found reliability improved by up to 10% compared with using separate RP. A joint Nordic Red dairy cattle RP was intended to improve the accuracy of genomic prediction in the previous study [15]. However, the results showed that the prediction for Swedish and Finnish population was improved slightly when the Danish Red dairy cattle were added into the RP since the relationship between Finnish Red and Swedish Red was closer compared with the relationship between Danish Red and the other two populations [15]. Similar pattern was observed when G matrix was used to measure the relationship among three countries in our study and the report from Brøndum et al. [15]. Higher related individuals were observed between Swedish and Finnish Red in their study and between American population and French population in our study. It is consisted with the conclusion from previous studies that the prediction ability was improved by including related individuals in the RP [16, 17]. The average of kinship among individuals from different countries was calculated, and the results showed the average relationship of any two countries was similar with the others (data not shown). One of the reasons could be that too many small values diluted the close relationship, which illustrated that the average of kinship matrix was not suitable as the criterion to measure the relationship between populations.

Another reason leading to the spearman’s rank correlation coefficient between GEBVs using Chinese RP and using American/French RP smaller than the other two correlations could be that the size of RP was different. Since Chinese RP in this study only included 1568 individuals, which may be not as informative as proven bulls from the other two countries. The combined RP between Nordic Holstein population, which is one member of Eurogenomics, and Chinese Holstein population had been utilized to investigate the improvement of reliability of genomic prediction in previous studies [5, 18]. The results showed the reliability of genomic prediction for Chinese population was improved greatly while little improvement for Nordic population [5]. Therefore, the size of RP should be considered when joint-population prediction was conducted besides taking the relationship between different populations into account. There is possibility to improve the genomic prediction ability for populations with a small number of RP even if the relationship between the added population and target population is distant.

## Conclusions

Information from the other related populations was applied to improve the predictive ability. However, our results showed that the GEBVs were in different rank when a loose related population was used as RP. Integrating results from previous studies, we concluded that it was feasible to predict the GEBVs of a target population using RP of a related population in the condition that there was a close genetic relationship between these two populations and the size of related population is large.

## Abbreviations

DRP: De-regressed proof
GEBVs: Genomic breeding values
LD: Linkage disequilibrium
PCA: Principal component analysis
